# (Four) Dual Plaquette 3D Ising Models

**DOI:** 10.3390/e22060633

**Published:** 2020-06-08

**Authors:** Desmond A. Johnston, Ranasinghe P. K. C. M. Ranasinghe

**Affiliations:** 1 School of Mathematical and Computer Sciences, Heriot-Watt University, Riccarton, Edinburgh EH14 4AS, UK; 2 Department of Mathematics, University of Sri Jayewardenepura, Gangodawila, Nugegoda 10250, Sri Lanka; ranasinghe@sjp.ac.lk

**Keywords:** statistical mechanics, Ising model, fractons

## Abstract

A characteristic feature of the 3d plaquette Ising model is its planar subsystem symmetry. The quantum version of this model has been shown to be related via a duality to the X-Cube model, which has been paradigmatic in the new and rapidly developing field of fractons. The relation between the 3d plaquette Ising and the X-Cube model is similar to that between the 2d quantum transverse spin Ising model and the Toric Code. Gauging the *global* symmetry in the case of the 2d Ising model and considering the gauge invariant sector of the high temperature phase leads to the Toric Code, whereas gauging the *subsystem* symmetry of the 3d quantum transverse spin plaquette Ising model leads to the X-Cube model. A non-standard dual formulation of the 3d plaquette Ising model which utilises three flavours of spins has recently been discussed in the context of dualising the fracton-free sector of the X-Cube model. In this paper we investigate the classical spin version of this non-standard dual Hamiltonian and discuss its properties in relation to the more familiar Ashkin–Teller-like dual and further related dual formulations involving both link and vertex spins and non-Ising spins.

## 1. Introduction

The Kramers–Wannier dual [1] of the classical Ising Hamiltonian with nearest neighbour 〈ij〉 couplings on a 3d cubic lattice

(1) $$H_{Ising}=-\sum_{\langle ij\rangle }\sigma_{i}\sigma_{j}$$

is the 3d Ising gauge theory

(2) $$H_{Gauge}=-\sum_{□}UUUU$$

where the sum runs over plaquettes □ and the gauge spins *U* live on the edges of the plaquettes. The coupling β in the partition function $Z\left(\beta \right)=\sum_{\left\{\sigma \right\}}\exp\left(-\beta H_{Ising}\right)$ and its dual $\beta^{*}$ in $Z\left(\beta^{*}\right)=\sum_{\left\{U\right\}}\exp\left(-\beta^{*}H_{Gauge}\right)$ are related by $\beta^{*}=-\frac{1}{2}\log\left[\tanh\left(\beta \right)\right]$. We use un-superscripted variables, e.g., $U,\sigma_{i},\tau_{i},\mu_{i}$, to denote spins in classical Hamiltonians and superscripted variables, e.g., $\sigma_{i}^{x,z},\tau_{i}^{x,z},\mu_{i}^{x,z}$, to denote the Pauli matrices appearing in quantum Hamiltonians. The positional subscript indices i,j,k… are occasionally omitted for brevity.

In this paper we will investigate the relation between four (apparently) different formulations of the dual to the 3d plaquette Ising model, which has also been dubbed the *gonihedric* Ising model [2,3,4,5]

(3) $$H_{\kappa =0}=-\sum_{□}\sigma_{i}\sigma_{j}\sigma_{k}\sigma_{l}\;.$$

This, like the 3d Ising gauge theory, has a plaquette □ interaction but the spins now reside at the vertices of a 3d cubic lattice rather than on its edges. The subscript κ=0 appears because this plaquette Hamiltonian is a particular case of a one-parameter family of gonihedric Hamiltonians

(4) $$H_{gonihedric}=-4\kappa \sum_{\langle ij\rangle }\sigma_{i}\sigma_{j}+\kappa \sum_{\langle \langle ij\rangle \rangle }\sigma_{i}\sigma_{j}-\left(1-\kappa \right)\sum_{□}\sigma_{i}\sigma_{j}\sigma_{k}\sigma_{l}\;.$$

defined by Savvidy and Wegner [6,7,8,9,10,11,12,13,14,15,16], where the 〈〈ij〉〉 are next-to-nearest neighbour spin interactions. The weights of spin cluster boundaries in this Hamiltonian are tuned to mimic a gas of worldsheets arising from a gonihedric string action. When the gonihedric string worldsheets are discretized using triangulations, their action may be written as

(5) $$S=\frac{1}{2}\sum_{\langle ij\rangle }\left|{\vec{X}}_{i}-{\vec{X}}_{j}\right|\;\theta \left(\alpha_{ij}\right),$$

where $\theta \left(\alpha_{ij}\right)=\left|\pi -\alpha_{ij}\right|$, $\alpha_{ij}$ is the dihedral angle between the neighbouring triangles with a common edge 〈ij〉 and $|{\vec{X}}_{i}-{\vec{X}}_{j}|$ are the lengths of the triangle edges. The ${\vec{X}}_{i}$ give the embeddings of the vertices *i* of the worldsheet discretization in the ambient spacetime.

The word gonihedric was originally coined to reflect the properties of this action, which weights edge lengths between non-coplanar triangles rather than the triangle areas, which is the case with a discretization of the standard Nambu–Goto/Polyakov string action. It combines the Greek words gonia for angle, referring to the dihedral angle, and hedra for base or face, referring to the adjacent triangles. $H_{gonihedric}$ is an appropriate cubic lattice discretization for such an action because it too assigns zero weight to the areas of spin cluster boundaries, weighting only edges and intersections [17]. This gives $H_{gonihedric}$ very different properties to $H_{Ising}$ where only the areas of spin cluster boundaries are weighted.

**Figure 1:**
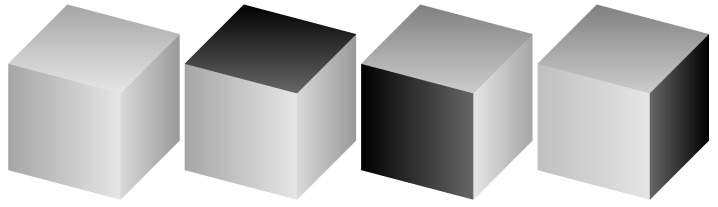
Flipping the value of the Ising spins on a face of a single cube in the 3d plaquette Ising Hamiltonian $H_{\kappa =0}$ does not change its contribution to the energy. The first cube configuration is the ferromagnetic state with all spins +. The spins at the corners of the dark shaded faces on the other three are −, the others +. All four of the single cube configurations shown have the same energy.

The 3d plaquette Ising action $H_{\kappa =0}$ has been shown to possess an exponentially (but sub-extensively) degenerate low-temperature phase and a first order phase transition as well as interesting, possibly glassy, dynamical properties [18,19,20,21,22,23,24]. A characteristic feature is that it displays a planar subsystem symmetry in which planes of spins may be flipped at zero energy cost, accounting for the degeneracy of the low temperature phase. This can be seen by looking at single cubes with a flipped face as in Figure 1 and using these to tile the lattice. Since multiple faces can be flipped on the cube, intersecting planes of flipped spins are also possible.

The degeneracy affects the finite size scaling behaviour at the first order transition [25,26,27,28,29], changing the universal $1/L^{3}$ finite size scaling shift in estimates of a first order transition point on an $L^{3}$ lattice (with periodic boundary conditions) [30,31] to $1/L^{2}$. For non-zero κ the planar subsystem symmetry appears to be broken at finite temperature [32,33] and the transition becomes second order. The Kramers–Wannier dual to $H_{\kappa =0}$ takes the form of an anisotropic Ashkin–Teller model [34]. It still possesses a planar subsystem symmetry and degenerate low temperature phase, so the modified finite size scaling at the first order transition is observed there also [25,26,27,28,29].

The subsystem symmetry in the quantum spin version of the 3d plaquette Ising model has recently been shown to be closely linked to the properties of the X-Cube model [35], which has become a paradigmatic model for the new and rapidly developing field of fractons, which are quasiparticles with restricted mobility in isolation. Some recent reviews of what is now a burgeoning fracton literature can be found in [36,37]. To see the role played by the subsystem symmetry in constructing the X-Cube model, first consider gauging the *global* $Z_{2}$ symmetry in the case of the 2d quantum transverse spin Ising model

(6) $$H=-\beta \sum_{\langle ij\rangle }\sigma_{i}^{z}\sigma_{j}^{z}-h\sum_{i}\sigma_{i}^{x}\;.$$

This can be done by introducing $\tau^{z}$ on the links and an additional plaquette flux term to endow the link spins with dynamics, which gives a gauge-invariant Ising (or $Z_{2}$ gauge–Higgs [38,39,40]) model

(7) $$\begin{array}{ccc} H & = & -\beta \sum_{\langle ij\rangle }\sigma_{i}^{z}\tau^{z}\sigma_{j}^{z}-h\sum_{i}\sigma_{i}^{x}-\beta_{p}\sum_{□}\tau^{z}\tau^{z}\tau^{z}\tau^{z} \end{array}$$

where we have dropped the link indices on the $\tau^{z}$ for conciseness. The gauge-invariant sector of the high temperature phase, β→0, of this model, where $\sigma_{i}^{x}\prod_{k\in +,i}\tau_{k}^{x}=1$ and *k* labels the four edges (+) incident to vertex *i*, gives Kitaev’s Toric Code model [41,42]

(8) $$\begin{array}{ccc} H & = & -h\sum_{i}A_{i}-\beta_{p}\sum_{□}B_{□}\;. \end{array}$$

We use the gauge invariance to trade $\sigma_{i}^{x}$ for $\prod_{k\in +,i}\tau_{k}^{x}$, leaving the mutually commuting terms

(9) $$A_{i}=\prod_{k\in +,i}\tau_{k}^{x},\;\;\;\;B_{□}=\prod_{i\in □}\tau_{i}^{z}\;.$$

and customarily set $h=\beta_{p}=1$. The Toric code displays topological order and has anyonic quasiparticle excitations.

On the other hand, gauging the *subsystem* symmetry of the 3d plaquette Ising model in a similar manner leads to the X-Cube model [35]. In this case, when we start with the quantum transverse spin 3d plaquette Ising model Hamiltonian

(10) $$H=-\beta \sum_{□}\sigma_{i}^{z}\sigma_{j}^{z}\sigma_{k}^{z}\sigma_{l}^{z}-h\sum_{i}\sigma_{i}^{x}$$

gauging the $Z_{2}$ subsystem symmetry requires inserting a $\tau^{z}$ which lives on the plaquettes

(11) $$\begin{array}{ccc} H & = & -\beta \sum_{□}\tau^{z}\sigma_{i}^{z}\sigma_{j}^{z}\sigma_{k}^{z}\sigma_{l}^{z}-h\sum_{i}\sigma_{i}^{x}\;+\;\ldots \end{array}$$

**Figure 2:**
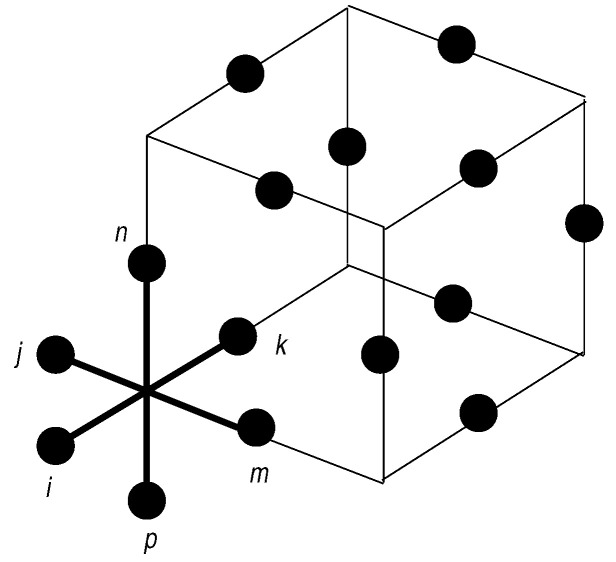
The terms contributing to the X-Cube Hamiltonian. The cube *A* term is a product of the twelve $\tau^{x}$ spins on the edges of the cube and the three *B* “X” terms composed of $\tau^{z}$ spins lie in each of the three lattice planes as shown on the corner.

The equivalent of the plaquette flux term in the Toric Code derivation is now a set of three “X” terms as shown in Figure 2, one in each lattice plane $B_{i}^{xy,yz,xz}=\prod_{j\in +,i}\tau_{j}^{z}$. If we again consider the gauge invariant sector $\;\;\sigma_{i}^{x}\prod_{k\in □,i}\tau_{k}^{x}=1$, where the $\tau_{k}^{x}$ live on the twelve incident plaquettes impacted by flipping the single central spin at site *i*, the high temperature limit β→0 produces the X-Cube Hamiltonian 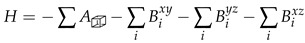 where it is simpler to think of the $\tau^{x},\tau^{z}$’s residing on the links of the dual lattice. The *A* term is a product of all the $\tau^{x}$ around a cube and the *B* terms are the three “crosses” of $\tau^{z}$ ’s shown in Figure 2.

The remaining couplings have again been set to one. The quasiparticles arising from defects in the *A* terms are fractons and cannot move in isolation, whereas the defects in the *B* terms give lineons, which can only move in straight lines. The order in the X-Cube model is not topological. It has an exponential, but sub-extensive, ground state degeneracy inherited from the plaquette Ising model as a consequence of the subsystem symmetry.

It was observed recently in [43] that the Hamiltonian for the fracton-free subsector (where all the *A* cube terms are +1) of the X-Cube model in a transverse field

(12) $$H=-\sum_{i}B_{i}^{xy}-\sum_{i}B_{i}^{yz}-\sum_{i}B_{i}^{xz}-g^{\prime }\sum \tau^{x}$$

could be written in terms of a dual Hamiltonian (at the risk of causing confusion we have kept the notation of [35] for the *A* and *B* terms rather than [43], which swaps *A* and *B*, though we denote the edge Pauli matrices by τ rather than σ in distinction to both [35,43]) with three flavours of Ising spins $\sigma_{i},\tau_{i},\mu_{i}$ living on the vertices of the cubic lattice rather than the links

(13) $$\begin{array}{cc} H= & -g^{\prime }\sum_{\langle ij\rangle }\sigma_{i}^{z}\sigma_{j}^{z}\mu_{i}^{z}\mu_{j}^{z}-g^{\prime }\sum_{\langle ik\rangle }\tau_{i}^{z}\tau_{k}^{z}\mu_{i}^{z}\mu_{k}^{z}-g^{\prime }\sum_{\langle jk\rangle }\sigma_{j}^{z}\sigma_{k}^{z}\tau_{j}^{z}\tau_{k}^{z}\\ & -\sum_{i}(\sigma_{i}^{x}\mu_{i}^{x}+\tau_{i}^{x}\mu_{i}^{x}+\sigma_{i}^{x}\tau_{i}^{x})\;, \end{array}$$

where the nearest neighbour sums in the four spin terms each run along one of the orthogonal axes, with ij,ik and jk representing the *z*, *y* and *x* axes respectively. The constraint on the *A* terms is automatically resolved by these spins.

In this paper we discuss the properties of the classical spin version of this Hamiltonian,

(14) $$H_{dual2}=-\sum_{\langle ij\rangle }\sigma_{i}\sigma_{j}\mu_{i}\mu_{j}-\sum_{\langle ik\rangle }\tau_{i}\tau_{k}\mu_{i}\mu_{k}-\sum_{\langle jk\rangle }\sigma_{j}\sigma_{k}\tau_{j}\tau_{k}\;,$$

dubbed $H_{dual2}$ for reasons to be explained in the next section. We shall see that it is closely related via a gauge-fixing to the Ashkin–Teller-like [44] Hamiltonian, $H_{dual1}$, constructed using the classical Kramers–Wannier duality from the 3d plaquette Ising model, as well as though a decoration transformation to a third Hamiltonian, $H_{dual3}$, which mixes edge and vertex spins. We find that the characteristic planar subsystem symmetry of the 3d plaquette Ising model is still present in $H_{dual1,2,3}$ and also that the interesting, possibly glassy, dynamical properties of the 3d plaquette model are also apparent in the duals. The Hamiltonians $H_{dual2,3}$ are already implicit in the discussion by Savvidy and Wegner in [45] in the context of the general framework for dualities [46] in spin models.

## 2. Duals Galore

**Figure 3:**
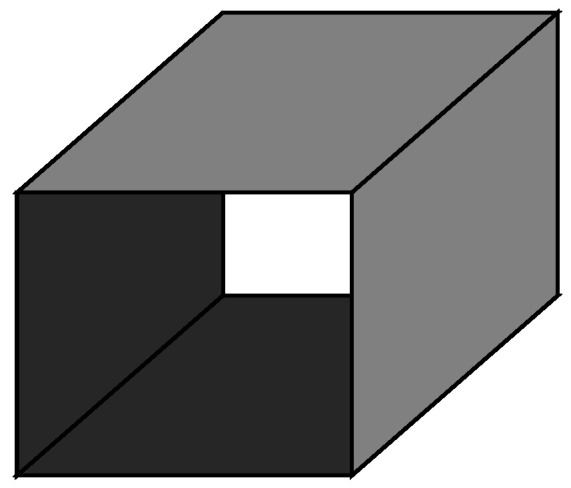
One of the matchbox surfaces which satisfy the algebra of Equation (18).

The Kramers–Wannier dual to $H_{\kappa =0}$ was initially constructed by Savvidy et al. [34] by considering the high temperature expansion of the plaquette Hamiltonian

(15) $$\begin{array}{cc} Z(\beta ) & =\sum_{\left\{\sigma \right\}}\exp\left(-\beta H_{\kappa =0}\right)\\ & =\sum_{\left\{\sigma \right\}}\prod_{□}\cosh(\beta )[1+\tanh(\beta )\left(\sigma_{i}\sigma_{j}\sigma_{k}\sigma_{l}\right)] \end{array}$$

which can be written as

(16) $$Z\left(\beta \right)={\left[2\cosh(\beta )\right]}^{3L^{3}}\sum_{\left\{S\right\}}{\left[\tanh(\beta )\right]}^{n(S)}$$

on an $L^{3}$ cubic lattice, where the sum runs over closed surfaces with an even number of plaquettes at any vertex. In the summation n(S) is the number of plaquettes in a given surface. The low temperature expansion, i.e., high temperature in the dual variable

$$\beta^{*}=-\left(1/2\right)\log\left[\tanh\left(\beta \right)\right]$$

of the following *anisotropic* Hamiltonian

(17) $$H_{dual0}=-\sum_{\langle ij\rangle }\sigma_{i}\sigma_{j}-\sum_{\langle ik\rangle }\tau_{i}\tau_{k}-\sum_{\langle jk\rangle }\eta_{j}\eta_{k}$$

produced the requisite diagrams. In $H_{dual0}$ the sums are one-dimensional and run along the orthogonal axes, with ij,ik and jk again representing the *z*, *y* and *x* axes respectively using our conventions. The spins are non-Ising and live in the fourth order Abelian group, since the geometric constraints on having an even number of plaquettes at each vertex mean that

(18) $$\begin{array}{ccc} e\sigma & = & \sigma \;,\;\;e\tau =\tau \;,\;\;e\eta =\eta \\ \sigma^{2} & = & \tau^{2}=\eta^{2}=e\\ \sigma \tau & = & \eta \;,\;\;\tau \eta =\sigma \;,\;\;\eta \sigma =\tau \end{array}$$

with *e* being the identity element. They can be thought of as representing differently oriented matchbox surfaces such as that shown in Figure 3, which are combined by facewise multiplication.

The shaded faces carry a negative sign and the associated spin variable lives at the centre of the matchbox. Any spin cluster boundary in the model can be constructed from such matchboxes while still satisfying the local constraint on the number of incident plaquettes.

The spins may also be taken to be Ising (±1) variables if we set $\eta_{i}=\sigma_{i}\;\tau_{i}$, which is more convenient for simulations. This modifies $H_{dual0}$ to an anisotropically coupled Ashkin–Teller Hamiltonian [44]

(19) $$H_{dual1}=-\sum_{\langle ij\rangle }\sigma_{i}\sigma_{j}-\sum_{\langle ik\rangle }\tau_{i}\tau_{k}-\sum_{\langle jk\rangle }\sigma_{j}\sigma_{k}\tau_{j}\tau_{k}\;.$$

This formulation of the dual model was first investigated numerically in [47] and it was found that it displayed a first order phase transition and a similar planar subsystem symmetry to that of $H_{\kappa =0}$. The continued presence of the subsystem symmetry was a consequence of the anisotropic couplings, which allowed a greater freedom in transforming the spin variables than in the isotropically coupled version of Equation (19), which is just the Ashkin–Teller model at its four-state Potts point.

It is possible to construct $H_{dual1}$ and its higher dimensional equivalents [45] using the general framework for duality in Ising lattice spin models that was first formulated by Wegner in [46]. Suprisingly, there are two further possible ways to write the dual to $H_{\kappa =0}$ in three dimensions with this machinery, using either the general formula for the dual of codimension one surfaces or the formula for the dual of two dimensional surfaces in *d* dimensions. If we temporarily use the notation of [45], the dual Hamiltonian for a codimension one surface in *d* dimensions is given there by

(20) $$\begin{array}{ccc} H_{dual,codim1}^{d} & = & -\sum_{\alpha <\beta ,\;\vec{r}}\prod_{\gamma }\Lambda_{\alpha ,\beta \gamma }\left(\vec{r}\right)\Lambda_{\alpha ,\beta \gamma }\left(\vec{r}+{\vec{e}}_{\gamma }\right)\Lambda_{\beta ,\alpha \gamma }\left(\vec{r}\right)\Lambda_{\beta ,\alpha \gamma }\left(\vec{r}+{\vec{e}}_{\gamma }\right) \end{array}$$

where the Λ spins live on each of the (d−3) dimensional (hyper)vertices situated at the vertices $\vec{r}$ of the hypercubic lattice and the indices α,β,γ run from 1 to *d*. The unit vectors ${\vec{e}}_{\gamma }$ point along the lattice axes. On the other hand, the dual Hamiltonian for a two-dimensional gonihedric surface embedded in *d* dimensions is of the form

(21) $$H_{dual,\;2d}^{d}=-\sum_{\vec{r}}\sum_{\beta \neq \gamma }\Lambda_{\beta \gamma }\left(\vec{r}\right)\Gamma \left(\vec{r},\vec{r}+{\vec{e}}_{\gamma }\right)\Lambda_{\beta \gamma }\left(\vec{r}+{\vec{e}}_{\gamma }\right)$$

where we now have Γ spins on each (hyper)edge in addition to the Λ spins at each vertex.

If we specialize to two dimensional plaquette surfaces embedded in a cubic lattice in three dimensions, which is the case for the dual of $H_{\kappa =0}$, either formulation may be employed since this is both a codimension one surface and a two-dimensional surface embedded in three dimensions. Returning to our own notation, the codimension one Hamiltonian of Equation (20) in three dimensions may be written as

$$H_{dual2}=-\sum_{\langle ij\rangle }\sigma_{i}\sigma_{j}\mu_{i}\mu_{j}-\sum_{\langle ik\rangle }\tau_{i}\tau_{k}\mu_{i}\mu_{k}-\sum_{\langle jk\rangle }\sigma_{j}\sigma_{k}\tau_{j}\tau_{k}\;,$$

which is just the Hamiltonian of Equation (14) that appeared as the classical spin limit of the dual to the fracton-free subspace of the X-Cube model. The three flavours of spins living at each vertex display a local Ising gauge symmetry $\sigma_{i},\tau_{i},\mu_{i}\to \gamma_{i}\sigma_{i},\gamma_{i}\tau_{i},\gamma_{i}\mu_{i}$ in addition to the planar subsystem symmetry shared with $H_{\kappa =0}$ and $H_{dual1}$, as we shall see presently.

Still within the general approach of Savvidy and Wegner [45,46], in three dimensions the Hamiltonian of Equation (21) for the two-dimensional surface variant also contains three flavours of vertex spins $\sigma_{i},\tau_{i},\mu_{i}$, but in addition there are gauge-like spin variables $U_{ij}^{1,2,3}$ living on the lattice edges which couple in an anisotropic manner to the vertex spins

(22) $$\begin{array}{cc} H_{dual3} & =-\sum_{\langle ij\rangle }(\sigma_{i}U_{ij}^{1}\sigma_{j}+\mu_{i}U_{ij}^{1}\mu_{j})-\sum_{\langle ik\rangle }(\tau_{i}U_{ik}^{2}\tau_{k}+\mu_{i}U_{ik}^{2}\mu_{k})\\ & -\sum_{\langle jk\rangle }(\sigma_{j}U_{jk}^{3}\sigma_{k}+\tau_{j}U_{jk}^{3}\tau_{k})\;. \end{array}$$

We thus have four different Hamiltonian formulations for the dual of the plaquette Hamiltonian $H_{\kappa =0}$ in three dimensions:

- $H_{dual0}$ in Equation (17) with non-Ising spins.
- $H_{dual1}$ in Equation (19) with Ising spins, which is Ashkin–Teller in form.
- $H_{dual2}$ in Equation (14) containing purely four spin interactions.
- $H_{dual3}$ in Equation (22) containing both vertex spins and gauge-like edge spins.

We have already seen that setting $\eta_{i}=\sigma_{i}\;\tau_{i}$ in $H_{dual0}$ in Equation (17), with $\sigma_{i},\tau_{i}$ being Ising spins, keeps the algebra of Equation (18) intact and gives the Ashkin–Teller Hamiltonian of $H_{dual1}$ in Equation (19). In the next section we discuss the relation between the four-spin Hamiltonian $H_{dual2}$ of Equation (14) and the gauge-spin Hamiltonian $H_{dual3}$ of Equation (22), and thereafter that between $H_{dual2}$ and $H_{dual1}$.

## 3. Decoration

**Figure 4:**
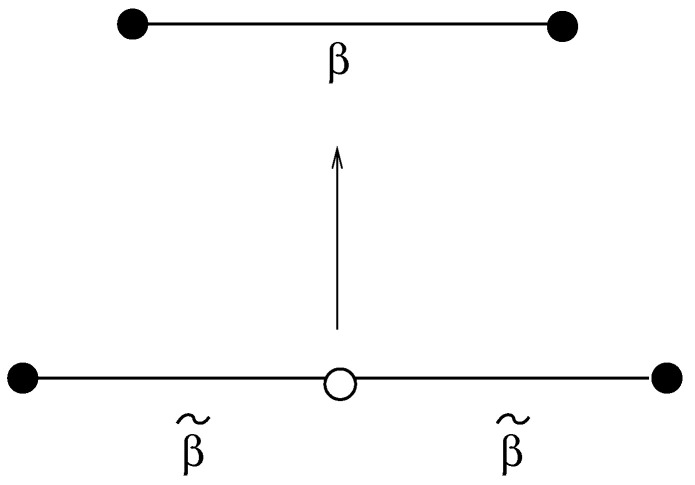
The standard decoration transformation. Summing over the central spin *s* denoted by an open dot gives a new effective coupling $\beta =\frac{1}{2}\log\left[\cosh\left(2\tilde{\beta }\right)\right]$ between the spins on the end of the link.

The equivalence between $H_{dual3}$ and $H_{dual2}$ is a consequence of a variation of the classical decoration transformation [48]. In the standard transformation an edge with spins $\sigma_{1},\sigma_{2}$ at each vertex is decorated with a link spin *s* as in Figure 4.

If the coupling between *s* and $\sigma_{1}$ and $\sigma_{2}$ is $\tilde{\beta }$, summing over the central spin *s* gives rise to a new effective coupling β between the primary vertex spins $\sigma_{1},\sigma_{2}$

(23) $$\sum_{s}\exp[\tilde{\beta }s\left(\sigma_{1}+\sigma_{2}\right)]=A\;\exp\left(\beta \sigma_{1}\sigma_{2}\right).$$

Both the prefactor *A* and the coupling β may be expressed in terms of $\tilde{\beta }$ by enumerating possible spin configurations in Equation (23). This gives

(24) $$\begin{array}{ccc} A & = & 2\cosh{\left(2\tilde{\beta }\right)}^{1/2}\\ \beta & = & \frac{1}{2}\log\left[\cosh\left(2\tilde{\beta }\right)\right]. \end{array}$$

We can repeat this procedure with the *U* spins on each edge in $H_{dual3}$. In this case each direction has two flavours of vertex spin and performing the sum generates the four-spin couplings of $H_{dual2}$, for example

(25) $$\begin{array}{c} \sum_{\left\{U_{12}^{1}\right\}}\exp[\tilde{\beta }(\sigma_{1}U_{12}^{1}\sigma_{2}+\mu_{1}U_{12}^{1}\mu_{2})]=A\;\exp\left(\beta \sigma_{1}\sigma_{2}\mu_{1}\mu_{2}\right). \end{array}$$

where *A* and the relation between $\beta ,\tilde{\beta }$ are the same as in Equation (24).

The sum over *U* may be carried out globally over every edge which immediately demonstrates equivalence of the partition functions for $H_{dual3}$ and $H_{dual2}$

(26) $$\begin{array}{cc} Z & =\sum_{\left\{U,\sigma \right\}}\exp\left[-\tilde{\beta }H_{dual3}\right]\\ & =\sum_{\left\{U,\sigma \right\}}\exp[\tilde{\beta }\sum_{\langle ij\rangle }(\sigma_{i}U_{ij}^{1}\sigma_{j}+\mu_{i}U_{ij}^{1}\mu_{j})+\tilde{\beta }\sum_{\langle ik\rangle }(\tau_{i}U_{ik}^{2}\tau_{k}+\mu_{i}U_{ik}^{2}\mu_{k})\\ & +\tilde{\beta }\sum_{\langle jk\rangle }(\sigma_{j}U_{jk}^{3}\sigma_{k}+\tau_{j}U_{jk}^{3}\tau_{k})]\\ & =B\;\sum_{\left\{\sigma \right\}}\exp[\beta (\sum_{\langle ij\rangle }\sigma_{i}\sigma_{j}\mu_{i}\mu_{j}+\sum_{\langle ik\rangle }\tau_{i}\tau_{k}\mu_{i}\mu_{k}+\sum_{\langle jk\rangle }\sigma_{j}\sigma_{k}\tau_{j}\tau_{k})]\\ & =B\;\sum_{\left\{\sigma \right\}}\exp\left[-\beta H_{dual2}\right]. \end{array}$$

The overall factor *B* coming from a product of *A*’s on the individual links is irrelevant for calculating physical quantities and the two couplings are again related by the decoration relation, $\beta =\frac{1}{2}\log\left[\cosh\left(2\tilde{\beta }\right)\right]$.

## 4. Gauge Fixing and Subsystem Symmetry

The equivalence between $H_{dual2}$ and $H_{dual1}$, on the other hand, is a consequence of a gauge symmetry which is present in $H_{dual2}$ [49]

(27) $$\sigma_{i},\;\tau_{i},\;\mu_{i}\;\to \;\gamma_{i}\sigma_{i},\;\gamma_{i}\tau_{i},\;\gamma_{i}\mu_{i}\;.$$

We are at liberty to choose the Ising spin gauge transformation parameter $\gamma_{i}$ to be equal to one of the spin values, say $\mu_{i}$, at each site so the gauge transformation then becomes

(28) $$\sigma_{i},\;\tau_{i},\;\mu_{i}\;\to \;\mu_{i}\sigma_{i},\;\mu_{i}\tau_{i},\;1$$

which, using the fact that the sum over the remaining spin variables $\sigma_{i},\tau_{i}$ is invariant under the transformation, relates the partition functions for the two Hamiltonians as

(29) $$\begin{array}{cc} Z & =\sum_{\left\{\sigma ,\tau ,\mu \right\}}\exp[-\beta H_{dual2}\left(\sigma ,\tau ,\mu \right)]\\ & =2^{L^{3}}\sum_{\left\{\sigma ,\tau \right\}}\exp[-\beta H_{dual2}\left(\sigma ,\tau ,\mu =1\right)]\\ & =2^{L^{3}}\sum_{\left\{\sigma ,\tau \right\}}\exp[-\beta H_{dual1}\left(\sigma ,\tau \right)]\;. \end{array}$$

The coupling β is not transformed in this case and we can, of course, choose to eliminate any one of the three spins, which simply amounts to relabelling the axes. From this perspective $H_{dual1}$ is simply a gauge-fixed version of $H_{dual2}$. This can be confirmed by Monte-Carlo simulations which measure the same energies (and energy distributions) and transition points for the observed first order phase transitions [49].

The equivalence between $H_{dual3}$ and $H_{dual2}$ described in the preceding section via the decoration transformation also sheds light on the presence of this gauge symmetry in $H_{dual2}$. All the terms in $H_{dual3}$ are of the gauge-matter coupling form $\sigma_{i}U_{ij}\sigma_{j}$, so this action possesses a similar, standard gauge invariance to that seen in other gauge-matter systems such as the gauge–Ising model of Equation (7), namely

(30) $$\begin{array}{c} \sigma_{i}\to \gamma_{i}\sigma_{i}\;,\;\sigma_{j}\to \gamma_{j}\sigma_{j}\;,\;U_{ij}^{1,3}\to \gamma_{i}U_{ij}^{1,3}\gamma_{j}\\ \tau_{i}\to \gamma_{i}\tau_{i}\;,\;\tau_{j}\to \gamma_{j}\tau_{j}\;,\;U_{ij}^{2,3}\to \gamma_{i}U_{ij}^{2,3}\gamma_{j}\\ \mu_{i}\to \gamma_{i}\mu_{i}\;,\;\mu_{j}\to \gamma_{j}\mu_{j}\;,\;U_{ij}^{1,2}\to \gamma_{i}U_{ij}^{1,2}\gamma_{j}\;. \end{array}$$

when the *U* spins are summed over to give $H_{dual2}$, the gauge symmetry of the σ,τ and μ spins in Equation (27) remains as an echo of this symmetry. In both cases if we look at a single site transformation all three spins $\sigma_{i},\tau_{i}$ and $\mu_{i}$ must be transformed. In $H_{dual3}$ this is a consequence of the way in which the three edge spins $U_{ij}^{1,2,3}$ couple to the vertex spins.

**Figure 5:**
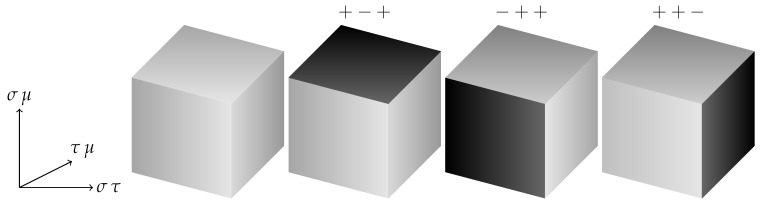
Four Possible ground state spin configurations on a cube for $H_{dual2}$. The initial cube again has all + spins and the σ,τ,μ values are shown for the spins at the corners of the darker shaded flipped faces, with the other spins being positive. The directions of the anisotropic couplings in the Hamiltonian are indicated.

A characteristic feature of $H_{dual1}$ is the planar subsystem symmetry intermediate between a gauge and a global symmetry, just as with the 3d plaquette Ising Hamiltonian. For $H_{dual2}$ the anisotropic couplings mean that it is still possible to flip planes of one of the spins (the one which is “missing” from the interactions in the direction perpendicular to the planes) at zero energy cost as shown in Figure 5.

It is also possible to flip two or three orthogonal faces on the cube, so tiling the entire lattice with such combinations we can see that in addition to the purely ferromagnetic ground state we may have arbitrary (and possibly intersecting) flipped planes of spins.

**Figure 6:**
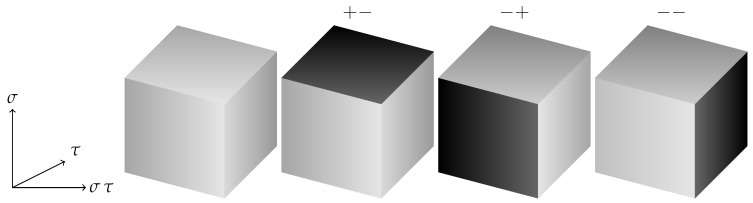
Four possible ground state spin configurations on a cube for the Ashkin–Teller formulation of the dual Hamiltonian, $H_{dual1}$. The σ,τ values are shown for the darker shaded flipped planes. The directions of the anisotropic couplings in the Hamiltonian are again indicated.

The ground state structure, and the mechanism of anisotropic couplings which allows the plane spin flips, is thus identical to that in $H_{dual1}$, whose possible ground states on a single cube we recall for comparison in Figure 6.

Flipping the third spin μ in $H_{dual2}$, which is absent in $H_{dual1}$, is replaced by flipping both the σ and τ spins in $H_{dual1}$, consistent with the gauge transformation relating the two Hamiltonians. In summary, the ground state structure of $H_{dual2}$ shows an interesting interplay between the gauge symmetry of Equation (27) and the subsystem symmetry. The local gauge symmetry allows one to reduce the effective number of degrees of freedom and recover the ground state structure of $H_{dual1}$.

$H_{dual3}$ is a similar case since the geometrical arrangement of the couplings is similar in spite of the presence of the additional spins *U* on the links. Each of the σ,τ,μ spins couple in two directions, which define the lattice planes in which they can be flipped without affecting the energy.

## 5. Indicative Monte-Carlo

**Figure 7:**
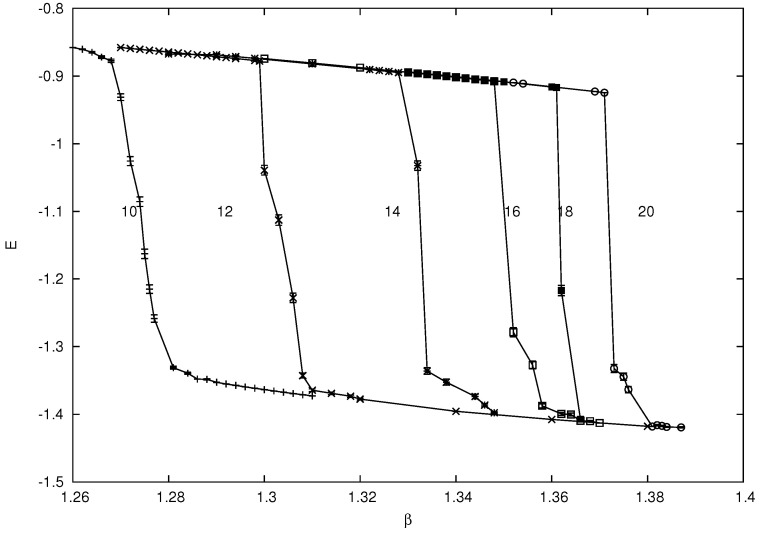
The energy for $H_{dual2}$ on lattices ranging from $10^{3}$ to $20^{3}$ from left to right. The lines joining the data points are drawn to guide the eye. Data from $H_{dual1}$ is essentially identical.

Low precision Monte-Carlo simulations using simple Metropolis updates found a first order phase transition in both $H_{dual2}$ and $H_{dual1}$ in the region β≃1.3−1.4 [47,49]. Much higher precision simulations using multicanonical methods were later carried out for the original 3d plaquette Ising model $H_{\kappa =0}$ and the Ashkin–Teller dual $H_{dual1}$ in order to accurately determine the transition point ($\beta^{\infty }=1.31328\left(12\right)$ in the case of the dual model) and confirm the non-standard finite size scaling that is a consequence of the exponential degeneracy of the low temperature phase [25,26,27,28,29]. Even with the modest statistics and the use of a Metropolis update in the simulations of [47,49] a sharp drop in the energy, as would be expected for a first order transition, is clearly visible in the region of the transition point. A plot of the energy is shown for various lattice sizes in Figure 7 for $H_{dual2}$ and the values for $H_{dual1}$ are essentially identical.

The first order nature of the transition for $H_{dual2}$ and $H_{dual1}$ can be further confirmed by observing a dual peak structure in the energy histogram P(E) near the transition point and a non-trivial value of Binder’s energy cumulant

(31) $$U_{E}=1-\frac{\langle E^{4}\rangle }{3{\langle E^{2}\rangle }^{2}}$$

as a consequence of the shape of P(E) [25,49].

Based on these observations, and allowing for a factor of 1/2 in our definitions of $H_{dual1}$ and $H_{dual2}$ in [47,49], we would expect to see a transition in $H_{dual3}$ at the the value of $\tilde{\beta }$ found by inverting the decoration transformation, namely $\frac{1}{2}\cosh^{-1}\left(\exp\left(1.3-1.4\right)\right)=0.99-1.04$ in the thermodynamic limit. To confirm this expectation, we carried out Monte-Carlo simulations using $10^{3},12^{3},16^{3}$ and $18^{3}$ lattices with periodic boundary conditions for all spins at various temperatures, again with a simple Metropolis update. After an appropriate number of thermalization sweeps, $10^{7}$ measurement sweeps were carried out at each lattice size for each temperature.

**Figure 8:**
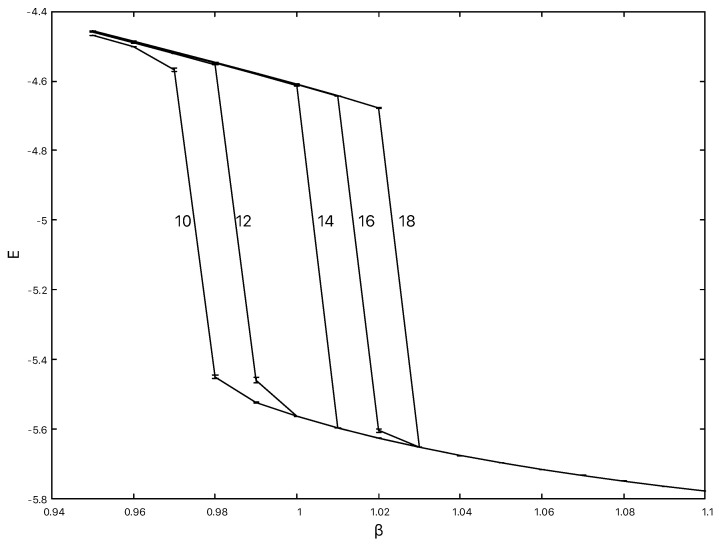
The energy for $H_{dual3}$ on lattices ranging from $10^{3}$ to $18^{3}$ from left to right. The lines joining the data points are drawn to guide the eye.

Looking at measurements of the energy from our simulations of $H_{dual3}$ in Figure 8 we can see that a similar sharp drop in the energy consistent with a first order transition is still present.

**Figure 9:**
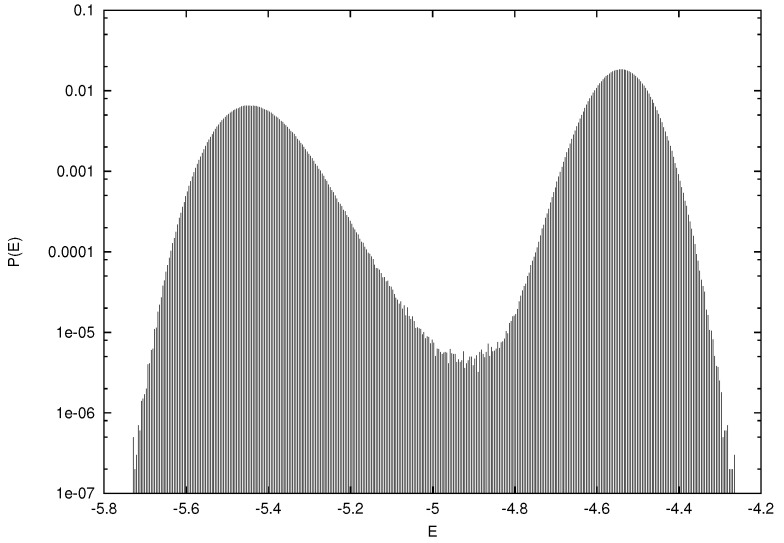
The energy histogram P(E) for $H_{dual3}$ close to the estimated transition point at β≃0.97 on a $10^{3}$ lattice.

The observed finite size estimates for the transition temperatures $\beta_{c}\left(L\right)$ agree with those calculated by transforming the values from Figure 7 using the decoration relation, e.g., for L=10 we would expect $\beta_{c}\left(10\right)≃\frac{1}{2}\cosh^{-1}\left[\exp\left(1.27\right)\right]≃0.97$, as found directly in the simulation shown in Figure 8. Further evidence for a first order transition with $H_{dual3}$, as noted above for the other dual Hamiltonians, can be garnered by looking at the energy histogram P(E) to discern a dual peak structure near the transition point. In Figure 9P(E) is shown close to the estimated transition point for L=10 at β≃0.97 and there is clear evidence of two peaks.

The relatively low statistics and the use of a Metropolis update for the data presented here for $H_{dual2}$ and $H_{dual3}$ mean that a high accuracy extrapolation using the correct $1/L^{2}$ finite size scaling for the transition point is not feasible, which would require more extensive multicanonical simulations along the lines of [25,26,29]. Nonetheless, the agreement of the suitably transformed finite size lattice transition points in the Monte-Carlo simulations confirm that $H_{dual3}$ and $H_{dual2}$ are related by the decoration transformation and the first order nature of the transition for $H_{dual3}$ is clear from the dual peak form of P(E) near the transition point in Figure 9.

## 6. Dynamics

Another interesting feature of the original plaquette Hamiltonian $H_{\kappa =0}$ is its dynamical behaviour. It possesses a region of strong metastability around the first order phase transition and displays glassy characteristics at lower temperatures [18,19,20,21,22,23,24] with non-trivial ageing properties. We found that the Ashkin–Teller dual Hamiltonian $H_{dual1}$ also shares these characteristics since it failed to relax to the equilibrium minimum energy of E=−1.5 when cooled quickly from a hot start [47]. Note that in this case, since we are exploring the real time dynamics of the system, simulations with a Metropolis update are preferable to more sophisticated algorithms.

**Figure 10:**
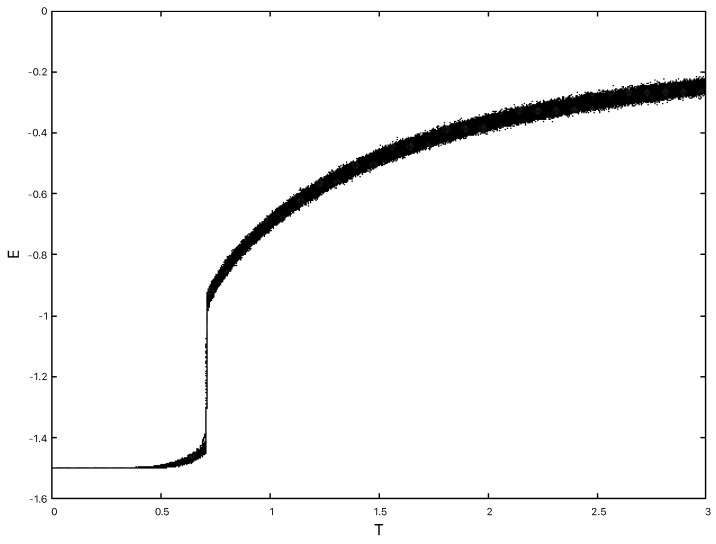
The time series of energy measurements obtained from cooling $20^{3},60^{3}$ and $80^{3}$ lattices from a hot start at T=3.0 at a rate of δT=0.00001 per sweep. The traces are effectively indistinguishable.

$H_{dual2}$ displays identical behaviour under cooling to $H_{dual1}$. We consider $20^{3},60^{3}$ and $80^{3}$ lattices which are first equilibrated in the high temperature phase at T=3.0 and then cooled at different rates to zero temperature. The energy time series is recorded during this process. In Figure 10 we can see that with a slow cooling rate of δT=0.00001 per sweep, the model still relaxes to a ground state with E=−1.5 for all the lattice sizes.

**Figure 11:**
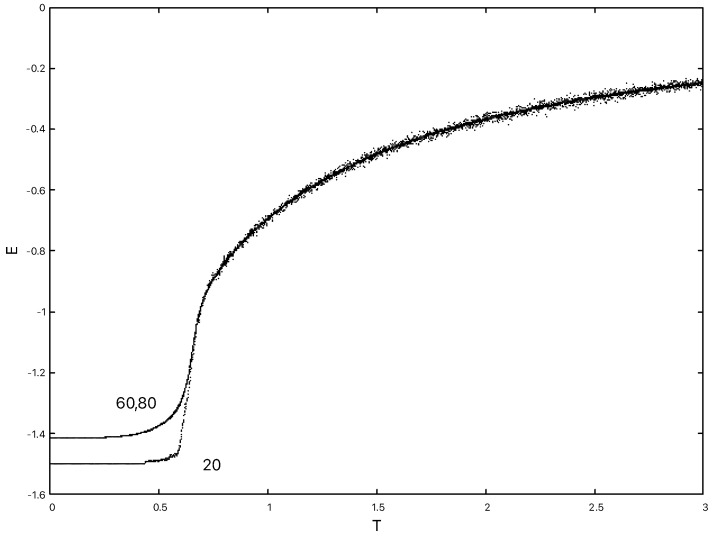
The time series of energy measurements obtained from cooling $20^{3},60^{3}$ and $80^{3}$ lattices from a hot start at a rate of δT=0.001 per sweep.

However, as can be seen in Figure 11 with a faster cooling rate of δT=0.001 per sweep the model no longer relaxes to the ground state energy of E=−1.5, but is trapped at a higher value, which is around −1.415 for the larger two ($60^{3}$ and $80^{3}$) lattices.

Whether the observed behaviour under cooling is a sign of genuine glassiness in $H_{\kappa =0}$ or not remains a matter of debate and similar considerations would apply to the dual Hamiltonians discussed here.

## 7. Discussion

Motivated by recent work on the quantum X-Cube Hamiltonian and related dual models [43] we revisit various formulations of classical spin Hamiltonians dual to the 3d plaquette Ising model. We describe the following chain of relations between these models

(32) $$\begin{array}{cc} H_{dual3}= & -\sum_{\langle ij\rangle }(\sigma_{i}U_{ij}^{1}\sigma_{j}+\mu_{i}U_{ij}^{1}\mu_{j})-\sum_{\langle ik\rangle }(\tau_{i}U_{ik}^{2}\tau_{k}+\mu_{i}U_{ik}^{2}\mu_{k})\\ & -\sum_{\langle jk\rangle }(\sigma_{j}U_{jk}^{3}\sigma_{k}+\tau_{j}U_{jk}^{3}\tau_{k})\\ & ⟶(\mathrm{Un})\mathrm{Decoration}⟶\\ H_{dual2}= & -\sum_{\langle ij\rangle }\sigma_{i}\sigma_{j}\mu_{i}\mu_{j}-\sum_{\langle ik\rangle }\tau_{i}\tau_{k}\mu_{i}\mu_{k}-\sum_{\langle jk\rangle }\sigma_{j}\sigma_{k}\tau_{j}\tau_{k}\\ & ⟶\mathrm{Gauge}-\mathrm{Fixing}⟶\\ H_{dual1}= & -\sum_{\langle ij\rangle }\sigma_{i}\sigma_{j}-\sum_{\langle ik\rangle }\tau_{i}\tau_{k}-\sum_{\langle jk\rangle }\sigma_{j}\sigma_{k}\tau_{j}\tau_{k}\\ & ⟶\mathrm{Non}-\mathrm{Ising}\mathrm{variables}⟶\\ H_{dual0}= & -\sum_{\langle ij\rangle }\sigma_{i}\sigma_{j}-\sum_{\langle ik\rangle }\tau_{i}\tau_{k}-\sum_{\langle jk\rangle }\eta_{j}\eta_{k}\\ & ⟶\mathrm{Kramers}-\mathrm{Wannier}\;\mathrm{duality}⟶\\ H_{\kappa =0}= & -\sum_{□}\sigma_{i}\sigma_{j}\sigma_{k}\sigma_{l} \end{array}$$

where we have indicated the operations relating the various Hamiltonians. A variant of the decoration transformation in which edge spins are summed out relates $H_{dual3}$ to $H_{dual2}$. In transforming $H_{dual3}$ to $H_{dual2}$ the coupling is therefore transformed as $\beta =\frac{1}{2}\ln\left[\cosh\left(2\tilde{\beta }\right)\right]$. The gauge-invariant nature of $H_{dual3}$ due to the presence of both edge and vertex spins leaves an echo in the vertex spin gauge symmetry of $H_{dual2}$, which in turn ensures the equivalence of $H_{dual2}$ and $H_{dual1}$ via a gauge-fixing. Allowing non-Ising spins gives a final equivalence between the dual models $H_{dual1}$ and $H_{dual0}$ and a standard Kramers–Wannier duality transformation then takes us back to the 3d plaquette Ising Hamiltonian of $H_{\kappa =0}$ where the story began.

The planar subsystem symmetry of this 3d plaquette Ising Hamiltonian $H_{\kappa =0}$ remains a feature of the various dual Hamiltonians and affects the finite size scaling properties at the first order transition displayed by these models, just as with $H_{\kappa =0}$. The nature of the order parameter for the various duals, and indeed $H_{\kappa =0}$ itself, remains to be satisfactorily clarified. An attempt at this has been made for $H_{\kappa =0}$ in [50] and indeed appeared to give sensible numerical results in [25,26,27,28,29]. This was based on the observation by Suzuki et al. [51,52,53] that an anisotropic version of the 3d plaquette Ising model with open boundary conditions could be transformed to an uncoupled stack of 2d Ising models and suggested that a two spin correlator summed perpendicular to lattice planes might still serve as an order parameter in the isotropically coupled case of $H_{\kappa =0}$. It would be more satisfactory to have a less heuristic approach to an order parameter based on a clearer understanding of the nature of the low temperature order in the 3d plaquette Ising Hamiltonian. In this respect a study of the order parameters in the various dual models might be helpful.

In a similar vein, the principal interest in [43] was actually investigating “odd” variants of fracton models, in which the signs of some of the terms in the Hamiltonians were reversed, leading to frustrated models. The geometrical nature of the order in such frustrated spin models would be of interest in the classical case too. Finally, the dynamics of the various classical Hamiltonians discussed here display glassy features. The question of whether the glassy dynamics of the quasiparticle excitations in the quantum models [54,55] offers any insights into this behaviour may be worth pursuing.
